# Secondary preventing effect of lung cancer in non-high-risk population: A retrospective investigation of opportunistic screening with low-dose computed tomography in Wuhan

**DOI:** 10.3389/fonc.2022.991485

**Published:** 2022-11-22

**Authors:** Zhiqiang Zhou, Chun Qiu, Shangkun Liu, Shaofang Wang, Danning Wang, Hui Xu

**Affiliations:** ^1^ Department of Anesthesiology, Tongji Hospital, Tongji Medical College, Huazhong University of Science and Technology, Wuhan, Hubei, China; ^2^ Lazaridis School, Wilfrid Laurier University, Waterloo, Ontario, ON, Canada; ^3^ Department of Nursing, Tongji Hospital, Tongji Medical College, Huazhong University of Science and Technology, Wuhan, Hubei, China; ^4^ Department of Radiology, Tongji Hospital, Tongji Medical College, Huazhong University of Science and Technology, Wuhan, Hubei, China

**Keywords:** chest LDCT, opportunistic screening, thoracic surgery, lung cancer, young female

## Abstract

**Background:**

Given the mortality benefit of low-dose computed tomography (LDCT) screening on high-risk populations, the retrospective investigation intended to identify the benefits of LDCT on lung cancer screening among the general demographic cohorts.

**Methods:**

We used an opportunistic screening with LDCT implemented during the pandemic in Wuhan to study the impact on subsequent thoracic surgeries, especially surgeries for lung cancer. Patients who received LDCT from October 1, 2019, to July 31, 2020, in three Triple-A accredited hospitals in Wuhan were included in the study. Relative week volumes of both surgeries before and after the chest LDCT screening were compared pairwise. The counts of surgeries for pulmonary nodules or masses, and corresponding pathological results among different gender and age groups were also compared.

**Result:**

The relative weekly volumes of thoracic surgery were significantly greater than those of stomach surgery after the opportunistic screening with LDCT. They were 33% (95% CI, 0.20-0.46; p<0. 001) higher than those of stomach surgery. For every 1,000 chest LDCT scans conducted in a given week, on average, 3.52(95% CI,0.56-6.49, p =0.03) thoracic surgeries were performed in the following week. After the implementation of opportunistic screening with LDCT, there was a higher percentage of young females with pulmonary nodule or mass (64.4% vs. 45.8%, p = 0.032). The fraction of lung cancer surgery in the treatment period was significantly greater than that in the control period (74.09% vs. 68.79%, p=0.007). There was a higher percentage of stage I lung cancer surgery in young and mid-age females than in the senior age group (64% vs. 53%, p= 0.05).

**Interpretation:**

Opportunistic screening with LDCT can advance the early diagnosis window of lung cancer in non-high-risk populations, especially young women who are easy to be ignored.

## Introduction

Despite healthcare progress and technological advances, Lung cancer remains one of the most common malignancies with the highest morbidity and mortality in China (1) and worldwide (2). However, Secondary prevention usually means early diagnosis with early treatment can significantly reduce mortality, as the five-year survival rate of patients with early lung cancer treatment exceeds 70% (3). Chest LDCT scans have been proven effective for early diagnosis. For example, yearly lung cancer screening for three years could reduce lung cancer mortality by 20% for heavy smokers (4). Nevertheless, to date, most early chest LDCT screenings are conducted among high-risk populations such as smokers or people with occupational exposure, and people with a family history of lung cancer. Routine chest LDCT scans to the general public, including younger demographics, are not offered in most countries, possibly due to the potential harms caused by lung cancer screening, such as false positive, radiation hazards, over-diagnosis, and excessive treatment (5–7). In addition, since lung cancer screening costs and health benefits vary from country to country, whether or not LDCT screening for lung cancer is cost-effective remains to be explored. Therefore, an opportunistic screening with LDCT implemented in Wuhan, China, to contain the pandemic among the general public provided a valuable opportunity to gauge the effects.

Accompanying the high volumes of chest LDCT scans is the post-pandemic rebound of thoracic surgeries in Wuhan. As the pandemic halted elective surgical services, it was expected that post-lockdown surgery volumes gradually return to the pre-pandemic level. Nevertheless, this was not the case for thoracic surgery. The volumes of thoracic surgery not related to the pandemic reached the pre-pandemic level in less than seven weeks. Furthermore, thoracic surgery continued to increase to 150% of the pre-pandemic level in nine weeks and stayed at 140% on average for another six weeks till July 2020. This puzzle motivated us to employ the setting of the large-scale chest LDCT scans as an opportunistic screening, under which we evaluated the value of *opportunistic screening with LDCT* in the early diagnosis of lung cancer. We hypothesized that the *opportunistic screening with LDCT* implemented in Wuhan could improve the early diagnosis window of lung cancer in non-high-risk populations.

## Methods

### Cohorts

We defined the period from October 2019 to January 2020 as the control period, and from April 2020 to July 2020 as the treatment period. The lockdown between these two periods, running from January to April 2020 was called interim period. In the corresponding period, patients who underwent thoracic surgery (lung/trachea, mediastinum, esophagus, pleural/chest wall) and stomach surgery were treated as the corresponding cohort.

### Natural experiment setting with the opportunistic screening

In this study, all thoracic surgeries conducted from October 2019 to July 2020 were included; stomach surgeries conducted during the same period were used as a comparison. It was expected that the relative weekly volumes of both types of surgeries should demonstrate similar patterns: plumped to zero due to the suspension of surgical services in the lockdown and climbed back to the pre-pandemic level in the treatment period. Nevertheless, we hypothesized that opportunistic screening with LDCT could improve the detection rate of thoracic diseases, especially the early diagnosis of lung cancer in the general population. Consequently, it is expected that, in the treatment period, thoracic surgeries not related to the pandemic shall return to or exceed the pre-pandemic level at a much faster pace than stomach surgeries.

### Data sources

**Figure 1:**
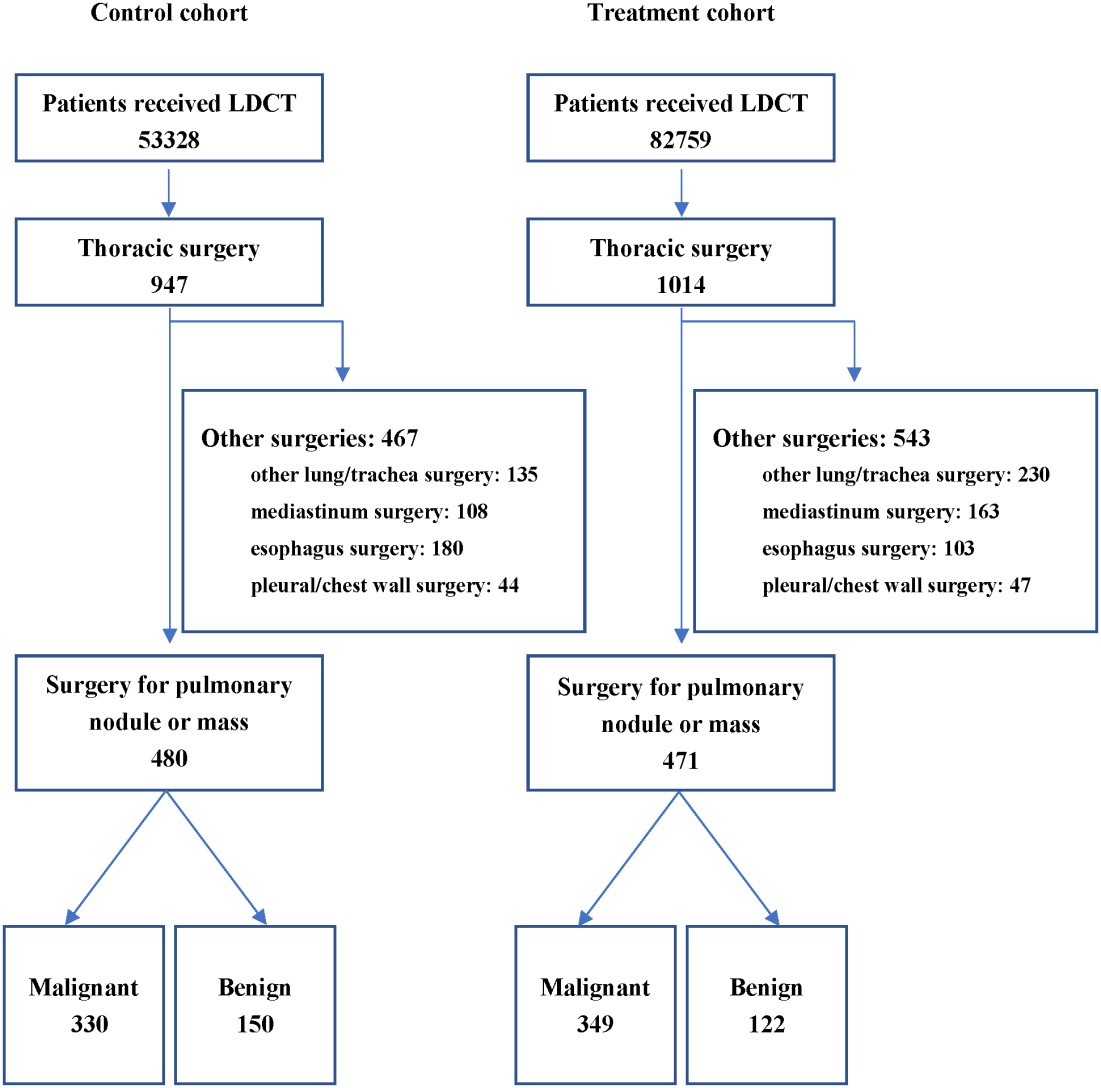
Flow Chart. The period from October 2019 to January 2020 was defined as the control period, and the period from April 2020 to July 2020 was defined as the treatment period. LDCT, low-dose computed tomography.

The study was approved by the Institutional Review Board of Tongji Hospital (TJ-IRB20210102). We have used data of surgeries and chest LDCT scans from the healthcare information system of three major Triple-A accredited hospitals in Wuhan, China, which jointly have more than 7,000 beds. We selected patients who received LDCT as the original study object. The surgical indication was determined by the thoracic surgeon and the patient. Patients who underwent stomach surgery during the same period were selected as the control (**Figure 1**). The following de-identified information: date of the surgery, surgery type (lung/trachea, mediastinum, esophagus, pleural/chest wall), and the duration of anesthesia and surgery; data of age, gender, body mass index (BMI), American Society of Anesthesiologists (ASA) physical status of the patients and the pathological classification were collected as well. Based on the individual data, we also aggregated the daily counts for each type of surgery from October 1, 2019, to July 31, 2020.

As the main object of this study, the surgical indications of patients with pulmonary nodules (solitary, larger part-solid nodules (≥6 mm) that presented with suspicious features (lobulated margins or cystic components), a developing solid component, or a large solid component (≥8 mm)) were strictly implemented according to the guidelines from the Fleischner society 2017 (8). Depending on whether it was a marginal pulmonary nodule or a central pulmonary nodule, these patients were performed marginal resection, segmental resection or lobectomy, respectively. All operations were performed with the prior consent of the patients. The stages of cancers were determined by pathological classification according to the eighth edition of the tumor-node-metastasis (TNM) classification (9). Stage Tis was confirmed as stage 0 lung cancer; stage groups of T1a-cN0M0 or T2aN0M0 were confirmed as stageI; T2bN0M0, T1a-cN1M0, T2a-bN1M0 or T3N0M0 as stageII; and T3N1M0,T4N1-2M0, or any TN2-3M0 as stage III.

### Outcomes

The primary outcome was the detection rate of LDCT in the opportunistic screening of lung cancer in the general population of different ages and genders. The secondary outcome was the effect of LDCT on the volume of thoracic operations. The relative weekly volumes of both types of surgeries. Daily counts were converted into weekly counts for each type of surgery and were further standardized by dividing the average weekly count of each surgery type of 2019. Therefore, the relative weekly volumes were presented as percentages, and 100% meant on par with the 2019 level, which was defined as the pre-pandemic level. The standardization accounted for the inherent differences in the magnitudes of surgery counts.

### Statistical analyses

We first compared the relative weekly volumes among two surgery types both in control and treatment periods using t test and Pearson χ² test. We then estimated the linkage between chest LDCT scans and thoracic surgery volumes using an autoregressive linear regression. We constructed a regression model on the weekly counts of thoracic surgery and the numbers of chest LDCT scans conducted some time earlier. Finally, we conducted subgroup comparisons based on gender, age, and pathology of surgery for pulmonary nodules or masses *via* Fisher’s exact test or Mann-Whitney test.

To present a complete picture of the data, we also provided summary results of individual patients who received one of the two types of surgery. The information showed as absolute values and percentages for discrete variables and mean (SD) or median (IQR) for continuous variables. Comparisons between categorical variables were made using χ² test or Fisher’s exact test. Comparisons between continuous variables were made using the student’s t-test or the Mann-Whitney test. All analyses were conducted using R software (version 4.0.2 for Windows). All p values were reported on two-sided tests, and results were statistically significant at p<0.05.

## Results

### Effect of LDCT screening on the volume of thoracic surgeries

**Table 1** summarizes the patient information during the control period (between October 1, 2019, and January 22, 2020) and the treatment period (between April 8 to July 31, 2020). In total, 1,238 patients received one of the two types of surgery (947 for thoracic surgery and 291 for stomach surgery) in the control period. 1,243 patients received one of the two types of surgery in the treatment period (1,014 for thoracic surgery, and 229 for stomach surgery). There was no significant difference in BMI, age distributions, or duration of anesthesia/operations between the control and the treatment periods (**Table 1**).

**Table 1:** Patient Characteristics In the Control and Treatment Periods.

		Control cohort	Treatment cohort	*P* value
**thoracic surgery**	**Age,** n/N, (%)			0.078
	<45 y	126/947, (13.31)	141/1014, (13.91)	
	45-60 y	427/947,45.09)	508/1014, (50.10)	
	60-75 y	370/947, (39.07)	340/1014, (33.53)	
	>75 y	24/947, (2.53)	25/1014, (2.47)	
	**Sex,** n/N, (%)			0.004
	Women,	401/947, (42.34)	497/1014, (49.01)	
	Men, n/N, (%)	546/947, (57.66)	517/1014, (50.99)	
	**BMI, median (IQR), (n),kg/m^2^**	23.02 (20.83,24.97), (944)	22.86 (20.80,24.97), (1010)	0.67
	**ASA physical status,** n/N, (%)			0.003
	I-II	771/947, (81.41)	769/1014, (75.84)	
	III-V	176/947, (18.59)	245/1014, (24.16)	
	**Emergency/Elective**, n/N, (%)			0.006
	Elective	815/947, (86.06)	914/947, (90.14)	
	Emergency	132/947, (13.94)	100, (9.86)	
	**Duration of anaesthesia, median (IQR), (n),min**	234.00 (188.00, 287.00), (947)	236.00 (190.00,295.00), (1014)	0.333
	**Duration of operation, median (IQR), (n),min**	177.00 (135.00, 230.00), (947)	176.50 (135.00,230.25), (1014)	0.68
**Stomach surgery**	**Age,** n/N, (%)			0.954
	<45 y	35/291, (12.03)	29/229, (12.66)	
	45-60 y	128/291, (43.99)	95/229, (41.48)	
	60-75 y	112/291, (38.49)	92/229, (40.17)	
	>75 y	16/291, (5.50)	13/229, (5.68)	
	**Sex,** n/N, (%)			
	Women,	95/291, (32.65)	68/229, (29.69)	0.532
	Men	196/291, (67.35)	161/229, (70.31)	
	**BMI, median (IQR), (n), kg/m^2^**	22.22 (19.98, 24.48), (289)	21.77 (19.49, 24.00), (225)	0.072
	**ASA physical status**, n/N, (%)			
	I-II	239/291, (82.13)	170/229, (74.24)	0.038
	III-V	52/291, (17.87)	59/229, (25.76)	
	**Emergency/Elective,** n/N, (%)			0.58
	Elective	274/291, (94.16)	219/229, (95.63)	
	Emergency	17/291, (5.84)	10/229, (4.37)	
	**Duration of anaesthesia, median (IQR), (n), min**	285.00 (223.00, 333.00), (291)	304.00 (213.00, 344.50), (229)	0.165
	**Duration of operation, median (IQR), (n), min**	240.00 (187.00, 290.00), (291)	255.00 (163.50, 304.00), (229)	0.378

Data are median (IQR), or n (%). BMI, body mass index. ASA, American Society of Anesthesiologists.

**Figure 2:**
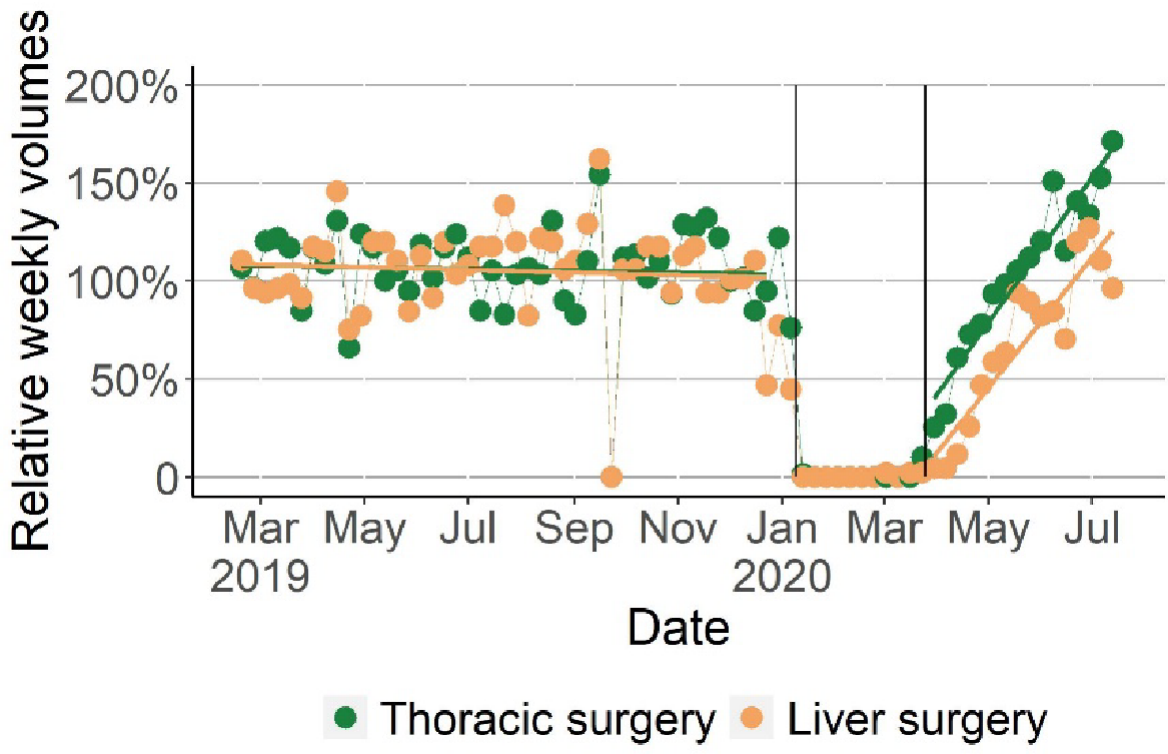
The Comparison of Changes in Relative Weekly Volumes of Thoracic Surgery with Stomach Surgery in the Control and Treatment Periods. Relative weekly volumes were the ratio of the weekly counts and the average weekly counts of surgery in 2019. The relative weekly volumes of thoracic surgery and stomach surgery were plotted respectively for the control and treatment periods. By the end of Treatment period (July 2020), the relative weekly volumes of thoracic surgery reached 150% of the weekly average of 2019.

**Figure 2**, which was plotted in a pairwise comparison manner, graphs the changes in the relative weekly volumes between thoracic surgery and stomach surgery. The relative weekly volumes of both types of surgery abruptly dropped to a very low level when the pandemic started and started to return to the pre-pandemic level in the control period when the disease was contained. Thoracic surgery experienced an exceptional surge that was much higher than stomach surgery: They returned to the 2019 average in mid-May 2020 and maintained the momentum. By the end of July 2020, the weekly thoracic surgery volume was 50% higher than the 2019 average. In comparison, the stomach surgery slowly recovered to the corresponding weekly averages of 2019 until the end of July 2020 (**Figure 2**). Then, we used a t-test to compare thoracic surgery volumes with that of stomach surgery in the control and treatment periods, respectively. The relatively weekly volumes of thoracic surgery did not differ significantly from that of stomach surgery in the control period. Nevertheless, in the treatment period, the relative weekly volumes of thoracic surgery were 33% higher than those of stomach surgery (95% CI, 0.20-0.46; p<0. 001) (**Table 2**). Furthermore, the regression analysis showed the dynamic association between the weekly volumes of chest LDCT and thoracic surgery in the treatment period. We found that the weekly volumes of thoracic surgery performed in week t were associated with chest LDCT volumes taken in week t-1 (one week earlier): for every 1,000 chest LDCT scans taken in week t-1, on average 3.52 (95% CI, 0.56-6.49; p=0.03) thoracic surgery was performed in the week that followed (**Table 3**).

**Table 2:** Pair-wise Comparison in Relative Weekly Volumes of two Surgery Types.

	Mean (SD) of relative weekly volumes by month, %^a^		t-statistic	*P* value
	Control cohort^b^	Treatment cohort^c^		
Thoracic surgery vs.	101 (31)	104 (42)	.	.
Stomach surgery	104 (30)	60 (30)	5.71 (0.01-0.47)	<0.001

a Relative weekly volumes were computed by dividing the weekly counts in the control period and treatment period the average weekly counts of the correspondingly surgery in 2019, respectively.

b Patients who received a surgery from October 1, 2019 to January 22, 2020.

c Patients who received a surgery from April 8, 2020 to July 31, 2020.

**Table 3:** Regression Results for the Impact of Absolute Count of Chest LDCT Scans in Week t-1 on thoracic Surgery in Week t.

	Mean (SD) of weekly counts		Estimated impact of every 1,000 chest LDCT scans on surgery counts (95% CI)^c^	*P* value
	Interim period^a^	Treatment period^b^		
Chest LDCT scans	2,923.18 (1,135.02)	6,366.06 (2,278.42)	.	.
Thoracic surgery	0.64 (1.80)	61.31 (24.91)	3.52 (0.56-6.49)	0.03

a Interim period was defined between control and treatment periods, and ran from January 23, 2020 to April 7, 2020.

b Treatment period was from April 8, 2020 to July 31, 2020.

c The coefficient estimate represented the effect of every 1,000 chest LDCT scans conducted on the increase of lung surgery in the following week.

### Effect of LDCT screening on early diagnosis of lung cancer in general population

More female patients received thoracic surgery in the treatment period [from 401(42%) to 497(49%), p<0.01] (**Table 1**). When combined with a subgroup analysis of age, we found that more females in the young age group received nodule or mass surgery in the treatment period than that in the control period [from 27 (45.8%) to 47(64.4%), p=0.032] (**Table 4**). In comparison, there was no significant gender difference among all age groups for stomach surgery.

**Table 4:** Summary of Gender Composition of Surgery for lung nodule or mass by Age Groups.

	Number of female patients		Number of male patients		*P* value
	Control cohort	Treatment cohort	Control cohort	Treatment cohort	
	(n=210)	(n=236)	(n=270)	(n=235)	
Young age group	27 (12.86)	47 (19.92)	32 (11.85)	26 (11.06)	0.032
(< 45 y)**, n (%)**				
Middle age group	97 (46.19)	122 (51.69)	110 (40.74)	115 (48.94)	0.332
(45-60 y)**, n (%)**				
Senior age group	86 (40.95)	67 (28.39)	128 (47.41)	94 (40.00)	0.781
(> 60 y)**, n (%)**				

**Figure 3:**
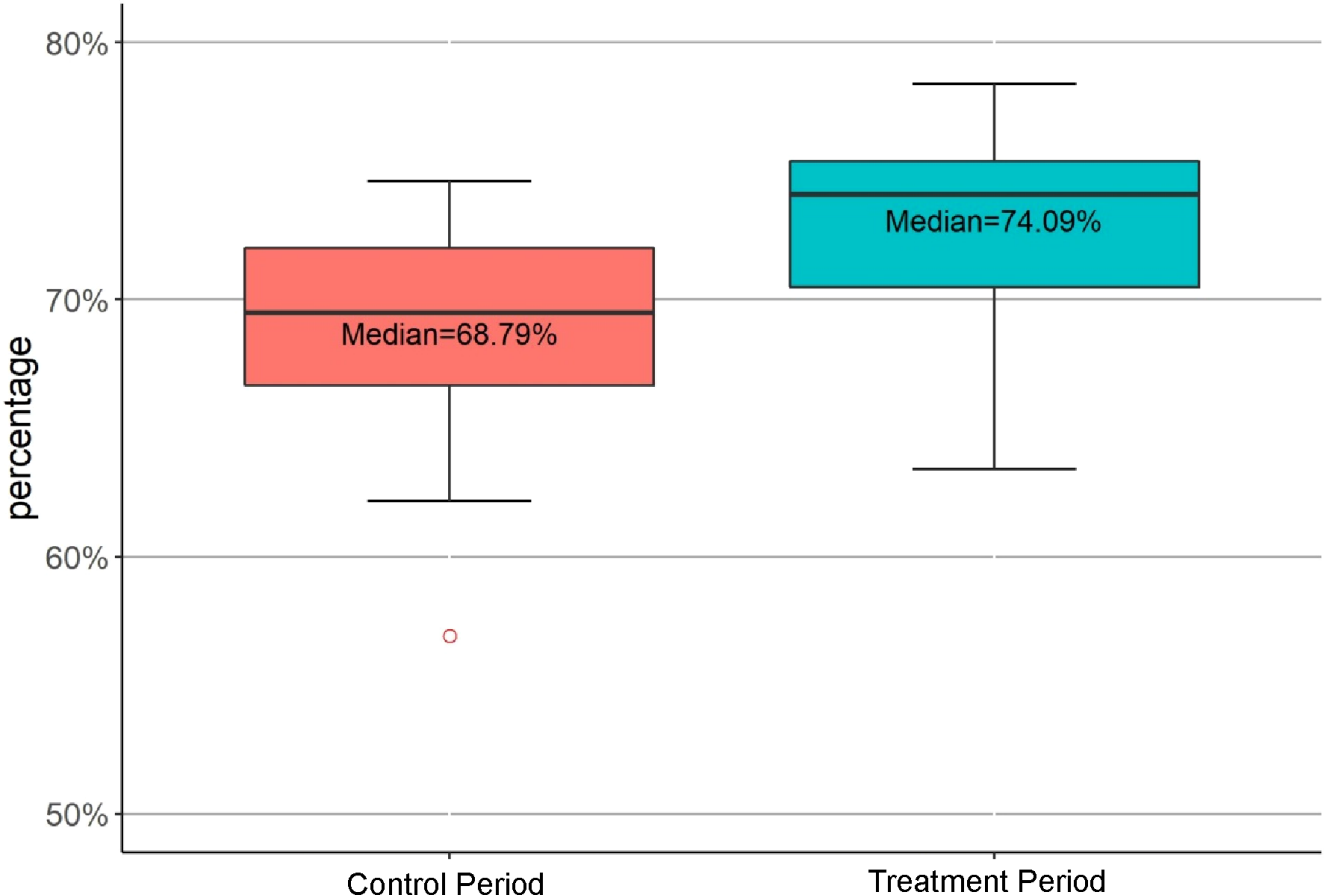
Comparison of the Fractions of Weekly Performed Lung Cancer Surgery in the Control and Treatment Periods. Percentages were the mean fractions of lung cancer surgery performed weekly. The mean fraction of lung cancer surgery in the treatment period was significantly greater than that of the control period (p=0.007).

For patients with pulmonary nodule or mass, according to the eighth edition of the TNM classification (9), the surgical records and pathological reports were further analyzed. In total, 679 patients were diagnosed with lung cancer. In the treatment period, the fraction of lung cancer surgery was significantly greater than in the control period (74.09% vs. 68.79%, p=0.007) (**Figure 3**). The clinical stages of 314 patients in the control cohort, and 337 patients in the treatment cohort were stage 0 or I. The counts of surgeries of different stages of lung cancer are summarized by age groups and gender (**Tables 5 A-D**). In the treatment period, there was a higher percentage of stage I patients in the young and mid-age female groups than that in the senior group [98 out 153(64%) vs.48 out 91 (53%), p= 0.05].

**Table 5:** A: Summary of Lung Cancer Composition by Gender and Age Groups Stage 0^a^.

	Control cohort		Treatment cohort		
	Females (n=67)	Males (n=43)	Females (n=64)	Males (n=29)	
Young age group (< 45 y)**, n (%)**	17 (25.4)	7 (16.3)	14 (21.9)	4 (13.8)	
Middle age group (45-60 y)**, n (%)**	29 (43.3)	22 (51.1)	34 (53.1)	19 (65.5)	
Senior age group (> 60 y)**, n (%)**	21 (31.3)	14 (32.6)	16 (25.0)	6 (20.7)	
^a^ Tis, carcinoma in situ. for the eighth edition of the TNM classification for lung cancer.					
**B: Summary of Lung Cancer Composition by Gender and Age Groups Stage I^b^**					
	**Control cohort**		**Treatment cohort**		
	**Females** **(n=105)**	**Males** **(n=99)**	**Females** **(n=146)**	**Males** **(n=98)**	
Young age group (< 45 y)**, n (%)**	5 (4.8)	5 (5.1)	15 (10.3)	11 (11.2)	
Middle age group (45-60 y)**, n (%)**	60 (57.1)	40 (40.4)	83 (56.8)	44 (44.9)	
Senior age group (> 60 y)**, n (%)**	40 (38.1)	54 (54.5)	48 (32.9)	43 (43.9)	
^b^ T1a-cN0M0 or T2aN0M0 for the eighth edition of the TNM classification for lung cancer.					
**C: Summary of Lung Cancer Composition by Gender and Age Groups Stage II^c^**				
	**Control cohort**		**Treatment cohort**	
	**Females** **(n=7)**	**Males** **(n=7)**	**Females** **(n=1)**	**Males** **(n=8)**
Young age group (< 45 y)**, n (%)**	0 (0)	0 (0)	0 (0)	0 (0)
Middle age group (45-60 y)**, n (%)**	4 (57.1)	3 (42.9)	1 (100)	3 (37.5)
Senior age group (> 60 y)**, n (%)**	3 (42.9)	4 (57.1)	0 (0)	5 (62.5)
^c^ T2bN0M0, T1a-cN1M0, T2a-bN1M0 or T3N0M0 for the eighth edition of the TNM classification for lung cancer.				
**D: Summary of Lung Cancer Composition by Gender and Age Groups Stage III^d^**					
	**Control cohort**		**Treatment cohort**		
	**Females** **(n=1)**	**Males** **(n=1)**	**Females** **(n=1)**	**Males** **(n=2)**	
Young age group (< 45 y)**, n (%)**	0 (0)	0 (0)	0 (0)	0 (0)	
Middle age group (45-60 y)**, n (%)**	1 (100)	1 (100)	1 (100)	2 (100)	
Senior age group (> 60 y)**, n (%)**	0 (0)	0 (0)	0 (0)	0 (0)	
^d^T3N1M0,T4N1-2M0, or any TN2-3M0 for the eighth edition of the TNM classification for lung cancer.				

## Discussion

Accounting for the increase due to schedule delays, we found that the surge in thoracic surgery was significantly higher than that in stomach surgery. The latter surgery also experienced a post-pandemic bounce, but the extent was significantly smaller than thoracic surgery. We also found a positive correlation between the volumes of thoracic surgery and chest LDCT scans, highlighting the importance of chest LDCT scans in thoracic diseases. It is well known that LDCT could advance the early diagnosis window of lung cancer and reduce the specific mortality of lung cancer. At the same time, LDCT can also detect potentially significant incidental findings (IFs) (10–12). In our study, the amount of thoracic surgery (including lung/trachea, mediastinum, esophagus, pleural/chest wall), other than pulmonary nodules, increased significantly in the treatment cohort. As stated in previous studies, the conduct of these findings will undoubtedly have a positive impact on the all-cause mortality of these patients (10–12).However, because the main purpose of this study was to evaluate the effect of opportunistic screening with LDCT on the early detection rate of lung cancer in the general population, we did not carry out a more in-depth investigation of these IFs.

We further probed into the composition of surgery for pulmonary nodules or masses. We found that the proportions of lung cancer surgery were higher in the treatment cohort than those in the control cohort (74.09% vs. 68.79% for lung cancer, p = 0.007). Two factors may contribute to this result. During the pandemic, there was a consensus to postpone surgery regardless of cancer type (13). The higher proportion of cancer operations in the treatment cohort would be partly due to the accumulation of delayed cases (14). Second, the high proportions were also closely related to the opportunistic screening with LDCT, which has helped early screening of patients with lung cancer, who were then given priority for surgical treatment.

How to combine daily medical services with target disease screening to improve the early diagnosis rate of lung cancer in the general population is a problem that needs to be considered in the current lung cancer screening. At present, most lung cancer screening guidelines recommend 55 as the starting age for lung cancer screening (15–17). The onset age of lung cancer in China is about five years earlier than that in Europe and the United States (18). In this study, more young female patients underwent lung nodule or mass surgery in the treatment period. More importantly, while there was no difference among gender compositions across different age groups in stage 0, we found a higher proportion of the young and mid-aged female patients who experienced stage I lung cancer in the treatment cohort. Usually, the incidence of lung cancer in women, especially in 18-45, was low. Therefore, lung cancer screening on this demographic group was conducted less frequently. As we know, passive smoking and long-term exposure to harmful indoor air pollution in the kitchen are high-risk factors for lung cancer among women. A study of 4,690 lung cancer screeners showed that although passive smokers accounted for only 20% of non-smoking women, lung cancer detection rate in this group was significantly higher than that in other groups (1.4% and 0.9%) (19). Another research showed that among non-smoking women, the risk of lung cancer in dust-exposed women was 2.47 times higher than that in non-exposed women (OR=2.47,95%CI:1.21~5.03) (20). The study suggests that young women with high-risk factors for lung cancer (smoking, environmental pollution, occupational exposure, previous chronic lung diseases (such as chronic obstructive pulmonary disease, pulmonary tuberculosis, pulmonary fibrosis and family history of tumor diseases) should also be included in the sufficient screening.

This present investigation revealed the value of such chest LDCT screening that they advanced the diagnosis window for occult asymptomatic thoracic diseases, especially for early lung cancer in non-high-risk populations. However, a sustainable screening strategy for lung cancer shall not overburden the health care system. In addition, in order to improve the early diagnosis rate of lung cancer, the awareness of early diagnosis and early treatment of lung cancer, and the initiative participation in cancer screening of the residents should be improved.

The limitation of this study is that, in analyzing the linkage between the opportunistic screening with LDCT and thoracic surgery surges, the study used aggregate CT screening data and could not pinpoint the strict causal relationship. It is highly desirable to analyze CT screening information on individual patients, if available, to link diagnosis details to surgical treatment. Combined with a large sample study of more centers would be needed to confirm our findings.

## Interpretation

In Conclusion, this study documented the dynamics of thoracic surgery and the linage to opportunistic screening with LDCT. This present study not only implied that the large-scale chest LDCT scans policy helped screen the pandemic, but also improved the early diagnosis rate of opportunistic screening for lung cancer in non-high-risk population. particularly for young women. To our knowledge, no high-quality study is currently available to define the optimal specific high-risk subpopulation as screening candidates in the Chinese population. It is worth considering to increase the screening rate of chest LDCT among younger women who tend to be overlooked, a valuable practice to improve the survival and prognosis of this demographic group with lung cancer. With full consideration of lung cancer screening programs’ benefits, hazards, and cost-effectiveness, public health services can integrate such a prevention-focused move to continue improving health care across the country.

## Data availability statement

The original contributions presented in the study are included in the article/supplementary materials. Further inquiries can be directed to the corresponding author.

## Ethics statement

The studies involving human participants were reviewed and approved by Institutional Review Board of Tongji Hospital. Written informed consent for participation was not required for this study in accordance with the national legislation and the institutional requirements.

## Author contributions

HX had full access to all of the data in the study and takes responsibility for the data integrity and the accuracy of the data analysis. ZZ, CQ, and SL are co-first authors. Concept and design: All authors. Acquisition, analysis, or interpretation of data: All authors. Drafting of the manuscript: HX. Critical revision of the manuscript for important intellectual content: All authors. Statistical analysis: All authors. Administrative, technical, or material support: HX. Supervision: ZZ, CQ and SL. All authors contributed to the article and approved the submitted version.

## Conflict of interest

The authors declare that the research was conducted in the absence of any commercial or financial relationships that could be construed as a potential conflict of interest.

## Publisher’s note

All claims expressed in this article are solely those of the authors and do not necessarily represent those of their affiliated organizations, or those of the publisher, the editors and the reviewers. Any product that may be evaluated in this article, or claim that may be made by its manufacturer, is not guaranteed or endorsed by the publisher.
